# Free intraperitoneal air in infected pancreatic necrosis with intraperitoneal rupture: A rare presentation of a complex diseases

**DOI:** 10.1002/ccr3.8958

**Published:** 2024-05-26

**Authors:** Segni Kejela, Genet Ager, Meklit Solomon Gebremariam

**Affiliations:** ^1^ Department of Surgery, College of Health Sciences Addis Ababa University Addis Ababa Ethiopia; ^2^ American Medical Center Addis Ababa Ethiopia

**Keywords:** acute medicine, critical care medicine, infectious diseases, surgery

## Abstract

**Key Clinical Message:**

Among the multitude of causes for acute abdomen patients presenting with free intraperitoneal air, one almost never finds infected pancreatic necrosis as one of the culprits. In patients with risk factors for acute pancreatitis presenting with generalized peritonitis with free intraperitoneal air, consideration should be given to this often deadly entity.

**Abstract:**

Acute pancreatitis is a morbid acute abdominal pathology that has been increasing in incidence in recent years. Most patients have a mild disease and treated medically, while a few proportion require interventional procedures. We present the case of a 39‐year‐old male patient who presented with progressive abdominal pain, vomiting, and yellowish discoloration of the eyes. The abdominal condition progressed to the point where clinical signs became consistent with generalized peritonitis and an x‐ray finding of free intraperitoneal air. The patient underwent exploratory laparotomy with intraoperative findings of intraperitoneal rupture of infected pancreatic necrosis with intraperitoneal purulent collection. He was managed with necrosectomy and discharged improved after intensive care and general ward stay.

## 1 INTRODUCTION

Acute pancreatitis is a morbid pathology that has been increasing in incidence over the last decade.^1^ This increase in incidence has been reported in most parts of the developed world by roughly 1%, although the data from low‐income countries are lacking.^2^ The etiologic factors cited as the most common causes for acute pancreatitis are gallstones and alcohol use disorder, but rarer etiologies like hypertriglyceridemia, trauma, infection, and procedures have also been reported.^3^ Although the most patients with the disease have a predictably mild course, about a fifth of patients face local and systemic complications, most common of which is acute pancreatic necrosis.^4^ Pancreatic necrosis follows the same pathogenesis of acute pancreatitis, with both mechanical ductal obstruction and enzymatic destruction causing progressive damage to the pancreatic and peripancreatic parenchyma, eventually leading to a saponified, devascularized area that is prone to infection.^5^ Close to a third of patients with pancreatic necrosis will develop infection of the necrotic pancreatic and peripancreatic tissue at some point over the course of their illness.^6^ Gastrointestinal bacterial translocation is the source of bacteria for infected pancreatic necrosis.^7^ This infectious complication doubles an already high mortality of pancreatic necrosis by causing sepsis and multiple organ failure.^8^ Almost all infected pancreatic necrosis cases are limited within the anatomic boundary of the lesser sac.^9^ Here we present a rare presentation of intraperitoneal rupture of infected pancreatic necrosis with resultant radiologically detected free intraperitoneal air, a case that has only been reported three times in the past.

## 2 CASE HISTORY/EXAMINATION

A 39‐year‐old male patient presented with a complaint of epigastric abdominal pain with associated nausea and vomiting of 1 day duration. He had similar, but less intense complaints 3 months prior, for which he was given an unspecified oral medication and had improvement until the current presentation. He has had a daily alcohol intake of over 10 drinks per day for the last 15 years; otherwise, he has no previously known medical comorbidities.

Initial physical examination showed vital signs were within the normal range. There was icteric sclera and abdominal examination revealed right upper quadrant and epigastric tenderness.

### 2.1 Differential diagnosis, investigations and treatment

White blood cell count was 19,500/mm^3^, with neutrophil predominance of 90%. The serum total and direct bilirubin levels were 7.1 and 5.3 mg/dL, respectively. Abdominal ultrasound showed multiple echogenic contents within the gallbladder, casting posterior acoustic shadow with a thickened gallbladder wall of 5.4 mm. In addition, there was dilation of intrahepatic biliary ducts. The sonographic diagnosis was acute calculous cholecystitis.

**FIGURE 1:**
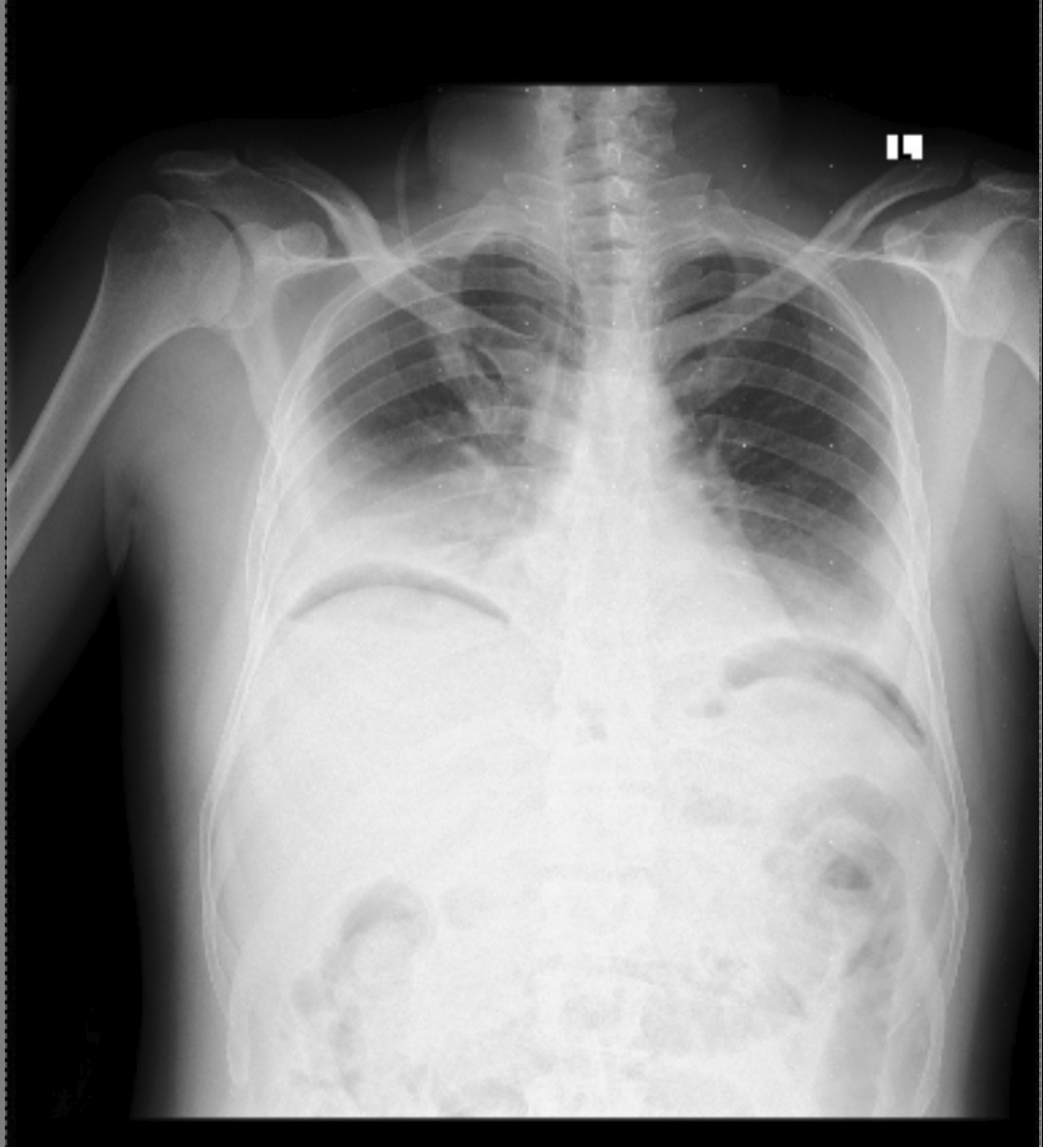
Erect chest x‐ray demonstrating air under the diaphragm with bilateral pleural effusion.

**FIGURE 2:**
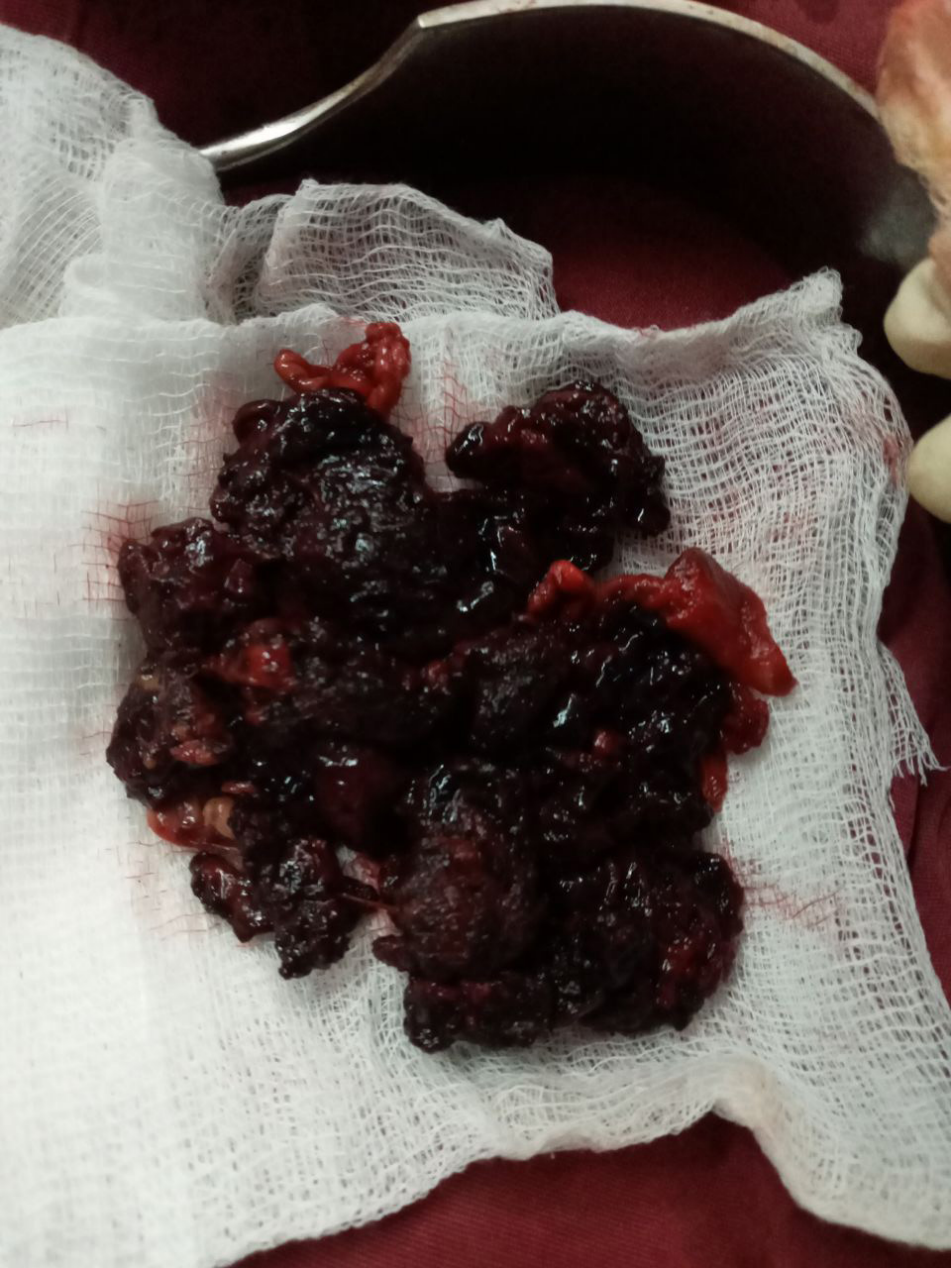
Necrosectomy of infected pancreatic necrosis containing necrotic and hemorrhagic content.

With an initial assessment of acute cholecystitis with acute cholangitis, the patient was initiated on ceftriaxone, metronidazole, diclofenac, and maintenance fluid as per the institutional standard. After a stable 36‐h course, he had an abrupt worsening of the abdominal pain with shortness of breath, high‐grade fever, and diaphoresis over a 6‐h period. During this time, he had associated nausea and vomiting of ingested matter of two episodes. On physical examination, he was in cardiorespiratory distress with a pulse rate of 132 bpm, a respiratory rate of 36/min, an oxygen saturation of 88% on 5 L of intranasal oxygen, a temperature of 38.7°C, and a blood pressure of 85/60 mmHg. He had icteric sclera with diffuse abdominal tenderness and distention. The erect chest x‐ray showed free intraperitoneal air under the diaphragm, as depicted on Figure 1. Assessment was consistent with generalized peritonitis secondary to viscus perforation, with differential diagnoses of perforated acute cholecystitis, perforated peptic ulcer disease, and perforated duodenal or gastric malignancy. With the goals of identifying the sight of perforation and providing definitive management, decision was made for emergency exploratory laparotomy. He was resuscitated with crystalloids and taken to the operating theater. The peritoneal cavity was accessed through a midline laparotomy incision, and the intraoperative findings were 1500 mL of hemorrhagic content mixed with purulent, foul‐smelling fluid in the peritoneal cavity, with a necrotic rupture through the hepatogastric ligament and pus draining into the peritoneal cavity from the lesser sac. Upon accessing the lesser sac through the greater omentum, the pancreas was found to be necrotic from the neck extending to the tail, with purulent content surrounding and within the necrotic tissue. In addition, there were multiple stones in the gallbladder with a dilated cystic and common bile duct. Pancreatic necrosectomy (Figure 2) with cholecystectomy and common bile duct exploration with transcystic tube drainage was performed. A 28 French chest tube was placed in the lesser sac, and a smaller subhepatic drain was inserted. The patient was transferred to the intensive care unit with septic shock, acute kidney injury secondary to septic acute tubular necrosis, respiratory failure, and disseminated intravascular coagulation. The patient was weaned off noradrenaline by the second postoperative day and extubated by the 6th postoperative day. The renal function test normalized from an initial creatinine value of 4.8 mg/dL by the 13th postoperative day. The patient had symptoms of alcohol withdrawal, which was diagnosed after extubation and improved after treatment with benzodiazepines.

### 2.2 Outcome and follow‐up

He was transferred to the general surgical ward on the 15th postoperative day. The transcystic, subhepatic, and lesser sac drains were removed by the 14th, 16th, and 24th postoperative days, respectively, and he was discharged on the 26th postoperative day. His outpatient clinic visit showed good tolerance to solid diet with no signs of malabsorption. He requires long‐acting insulin for glycemic control and has been able to perform routine daily functions independently.

## 3 DISCUSSION

The most common causes of pneumoperitoneum in patients presenting with signs of peritonitis are viscus perforations at the gastroduodenal, small bowel, appendiceal, and colorectal anatomic regions.^10^ Fewer patients present with perforation of the gallbladder from complications of acute cholecystitis.^11^ External causes from iatrogenic and traumatic incidents have also been reported.^12^, ^13^ The exact mechanism of pneumoperitoneum in infected pancreatic necrosis has not been described, but our conjecture is that it could be from either an undetected bowel fistula or infection from a gas‐producing organism.

There are three reports of free intraperitoneal air attributed to infected pancreatic necrosis in literature. Two of these reports demonstrated a CT scan diagnosis of pancreatic infected necrosis with free intraperitoneal air, while a single report depicted a similar finding of air under the diaphragm on an erect radiograph.^14^, ^15^, ^16^ CT was deferred in our case because of the need for immediate surgery in a patient clearly demonstrating generalized peritonitis with free intraperitoneal air seen on a radiograph. Only one patient had hemodynamic instability preoperatively and was weaned off pressors on the 2nd postoperative day.^14^ None of the patients had causes of acute pancreatitis reported. The culprit bacterial agents were reported in two of the studies as *Klebsiella* and *Clostridium perfringens*.^15^, ^16^ The type of antibiotics utilized was only reported for one case with *Klebsiella* infection isolated, who was treated with aminoglycosides.^14^ All three patients underwent necrosectomy/debridement and drainage.^14^, ^15^, ^16^ Double‐tube irrigation drainage was used in two of the patients reported.^14^, ^15^ We were confident enough in the extent of our debridement that we only utilized one large bore drainage for the lesser sac tube and a separate subhepatic tube for the cholecystectomy site drainage.^14^, ^15^

One death was reported in the patient with *Clostridium perfringens* infection who underwent a total abdominal colectomy with the assumption of an undetected perforation, which was not found on the postoperative biopsy.^16^

In the era of the step‐up approach of management of infected pancreatic necrosis, most cases are treated using either percutaneous or endoscopic approaches with a high success rate and lower morbidity.^17^ Most patients in high‐resource settings are treated in order of antibiotics first, followed by percutaneous drainage, then endoscopic procedures, minimally invasive surgery, and open surgery as a last resort.^17^ There are, however, circumstances that would render a surgeon to a decision for exploratory laparotomy. The main instance, as seen in the previously reported cases and ours, is the suspicion for perforated viscus.^14^, ^15^, ^16^ In this situation, it would be imperative not to delay surgical diagnosis and intervention. It is worth noting that laparoscopic approaches were utilized in one patient who's already had a CT scan diagnosis. As demonstrated by Kanchana and colleagues, laparoscopic surgical options are still possible in performing drainage and necrosectomy for intraperitoneal rupture of infected pancreatic necrosis.^14^ An additional factor influencing the method of intervention could be the institutional expertise and resources. In low‐income institutions like ours, the only available option is open surgery. Nonetheless, surgery is not without complications. Necrosectomies, especially when performed within 4 weeks of illness, are associated with high mortality rates.^18^ In addition, higher rates of perforated viscus, organ dysfunction, wound infection, and bleeding were reported in the open necrosectomy cohort as compared to the step‐up approach.^4^ However, in selected group of patients like ours, the benefit of open surgery is still confirmed in the modern surgical literature.^19^

An earlier investigation with cross‐sectional imaging and serum pancreatic enzymes could have led to an earlier diagnosis in our patient's case. Early diagnosis can prevent organ dysfunction and hemodynamic instability, which reduces the critical care unit stay of the patient and mortality.^20^

## 4 CONCLUSION

In conclusion, free intraperitoneal air is a rare presentation of infected pancreatic necrosis. Such patients can demonstrate peritoneal signs and signs of obstructive jaundice, narrowing the differential diagnosis to peritonitis secondary to perforation of biliary structures and pancreatic necrosis. These patients require urgent surgical intervention, which could entail drainage and necrosectomy of the infected necrosis. Earlier detection before hemodynamic instability ensues is important, as pancreatic necrosis patients tend to have a complicated course, and a delay in diagnosis and management of complications could worsen the prognosis further.

## AUTHOR CONTRIBUTIONS

**Segni Kejela:** Conceptualization; data curation; formal analysis; investigation; methodology; resources; writing – original draft. **Genet Ager:** Conceptualization; data curation; investigation; resources; writing – review and editing. **Meklit Solomon Gebremariam:** Conceptualization; data curation; investigation.

## FUNDING INFORMATION

No funding was acquired for this case report.

## CONFLICT OF INTEREST STATEMENT

The authors declare no conflicts of interest.

## ETHICS STATEMENT

This manuscript was written according to the world medical association Declaration of Helsinki.

## CONSENT

Written informed consent was obtained from the patient to publish this report in accordance with the journal's patient consent policy.

## Data Availability

Data for this case report would be available upon reasonable request to the authors.
